# Identification of Prognostic Immune-Related Genes by Integrating mRNA Expression and Methylation in Lung Adenocarcinoma

**DOI:** 10.1155/2020/9548632

**Published:** 2020-07-09

**Authors:** Jie Zhu, Min Wang, Daixing Hu

**Affiliations:** ^1^Department of Intensive Care Unit, The People's Hospital of Tongliang District, Chongqing, China; ^2^Department of Respiratory and Geriatrics, Chongqing Public Health Medical Center, Chongqing, China; ^3^Department of Urology, The First Affiliated Hospital of Chongqing Medical University, Chongqing, China

## Abstract

**Background:**

There is plenty of evidence showing that immune-related genes (IRGs) and epigenetic modifications play important roles in the biological process of cancer. The purpose of this study is to establish novel IRG prognostic markers by integrating mRNA expression and methylation in lung adenocarcinoma (LUAD).

**Methods and Results:**

The transcriptome profiling data and the RNA-seq data of LUAD with the corresponding clinical information of 543 LUAD cases were downloaded from The Cancer Genome Atlas (TCGA) database, which were analyzed by univariate Cox proportional regression and multivariate Cox proportional regression to develop an independent prognostic signature. On the basis of this signature, we could divide LUAD patients into the high-risk, medium-risk, and low-risk groups. Further survival analyses demonstrated that high-risk patients had significantly shorter overall survival (OS) than low-risk patients. The signature, which contains 8 IRGs (S100A16, FGF2, IGKV4-1, CX3CR1, INHA, ANGPTL4, TNFRSF11A, and VIPR1), was also validated by data from the Gene Expression Omnibus (GEO) database. We also conducted analyses of methylation levels of the relevant IRGs and their CpG sites. Meanwhile, their associations with prognosis were examined and validated by the GEO database, revealing that the methylation levels of INHA, S100A16, the CpG site cg23851011, and the CpG site cg06552037 may be used as the potential regulators for the treatment of LUAD.

**Conclusion:**

Collectively, INHA, S100A16, the CpG site cg23851011, and the CpG site cg06552037 are promising biomarkers for monitoring the outcomes of LUAD.

## 1. Introduction

According to the latest statistics in 2019, lung cancer still ranked first with regard to the different kinds of cancer mortality in the United States [1]. More than half (57%) of lung cancer patients are diagnosed at the later stages [2]. Even patients who underwent surgical resection, chemotherapy, radiotherapy, and targeted therapy did not have significantly improved survival. The five-year survival varies from 4 to 17%, leading to a need to explore new therapeutic targets [2, 3]. Lung cancer mainly has two subtypes, non-small cell lung cancer (NSCLC) and small cell lung cancer (SCLC). Lung adenocarcinoma (LUAD) and squamous cell carcinoma are the two main types of NSCLC, accounting for 40% of cases [4]. Molecular targeting therapies significantly improved prognosis in patients with LUAD. Tyrosine kinase inhibitors (TKIs) targeting the epidermal growth factor receptor (EGFR) served as a first-line treatment option for advanced LUAD with sensitizing EGFR mutation [5]. ROS protooncogene 1 (ROS1) and anaplastic lymphoma kinase (ALK) gene rearrangements are also common therapeutic targets for LUAD [6]. However, there are still a large number of mutation-negative patients, for which cancer immunotherapy has attracted considerable attention in recent years because the immune response in the tumor microenvironment is now recognized as a significant factor that determines tumor aggressiveness and progression. The development of immune checkpoint blockade therapy has been proven to achieve durable, long-term responses in lung cancers [7, 8].

Under regular conditions, tumor cells produce specific antigens, which are identified by antigen-presenting cells (APCs) to process tumor antigens and are combined with major histocompatibility complexes (MHC) 1 and 2 to express antigens on the surface of APCs. Presenting them to T cells and activating them to produce effector T cells conduct normal immune surveillance and avoid tumor production. However, tumor cells can escape immune surveillance and immune clearance through various factors. By loss of tumor antigenicity, possibly due to antigen processing presentation defects or MHC subunit presentation antigen defects, tumor immunogenicity is reduced. Besides, mutations in oncogenes and tumor suppressor genes lead to malignant cell transformation while recruiting inflammatory cells to induce a special immune response to create an immunosuppressive microenvironment to help escape immune clearance [9]. Antibodies against immune checkpoints like programmed death 1 (PD-1) and cytotoxic T lymphocyte-associated antigen-4 (CTLA-4) could be an effective potential treatment and demonstrate a remarkable, durable response in NSCLC [10, 11]. But the molecular characteristics describing tumor-immune interaction remain to be comprehensively explored regarding their prognostic potential in NSCLC.

Our efforts concentrated on developing an immune signature with prognostic ability based on the comprehensive list of IRGs downloaded from the Immunology Database and Analysis Portal (ImmPort) database. The RNA sequencing (RNA-seq) data and the microarray data from TCGA database and the GEO database were used for analysis. By multivariate Cox regression analysis, we obtained independent IRGs associated with the prognosis of LUAD. Then, we evaluated whether this signature was associated with the survival outcome of subgroups of LUAD patients and clinicopathological factors. The methylation levels of the relevant IRGs and their CpG sites were also analyzed, and their associations with prognosis were examined. We further validated our results in the GEO database, thus revealing that the methylation levels of IRGs and their CpG sites also significantly affected LUAD prognosis.

## 2. Materials and Methods

### 2.1. Samples and Data Extraction

Level 3 raw counts of the transcriptome profiling data and RNA-seq data of LUAD with corresponding clinical information of 479 cases were downloaded from TCGA database. Accordingly, the methylation data (beta values) of 543 cases with LUAD, which include lung tumor tissues and matched nontumor tissues, were collected on 8 December 2019. The GSE37745 was another transcriptome profiling data of 196 LUAD patients downloaded from the GEO database (https://www.ncbi.nlm.nih.gov/geo), which was used as the testing set for the prognostic IRG model. In addition, the GSE63384 dataset and the GSE83845 dataset were profiled using the Illumina Human Methylation 450 platform and used to validate the differential methylation levels of IRGs. The comprehensive list of IRGs containing a total of 2499 genes was downloaded from the ImmPort database (https://immport.niaid.nih.gov), including antigen processing and presentation pathways, cytokines, cytokine receptors, T-cell receptor signaling pathway, B-cell antigen receptor signaling pathway, and natural killer cell cytotoxicity. For genes with multiple probes, the average value was used as their expression values.

### 2.2. Statistical Analysis

Differentially expressed genes were screened using the package limma in the R program 3.6.3. The survival analysis was performed by the package survival. A risk scoring system was established via univariate Cox regression and multivariate Cox regression through the R program. After classifying LUAD patients into subtypes of low-risk, medium-risk, and high-risk, principal component analysis (PCA) was used to evaluate the effectiveness of classification. To predict survival by the Kaplan-Meier method with hazard ratios (HR) calculated, the package survival and the package survminer were performed in the R program and GraphPad software (Prism 8). Univariate Cox regression was also used to analyze the clinical features and the risk score for association with overall survival (OS). Multivariate Cox regression analysis indicated its independent prognostic value. The prediction accuracy of the risk system was determined by time-dependent Receiver Operating Characteristic (ROC) analysis. We carried out a series of gene functional enrichment analyses to determine the major biological attributes, including the GO and KEGG analyses. The GOplot package was employed to visualize the enrichment terms. Chi-square test for parametric distributions or the Wilcoxon test for nonparametric distributions was used. We considered *P* < .05 significant for all comparisons.

### 2.3. Identification of Differentially Expressed mRNAs (DEGs) and IRG Model in LUAD and Adjacent Normal Tissues

To identify DEGs between tumor tissues and adjacent normal tissues, we performed differential expression analysis using the limma package of R software. The thresholds for screening DEGs were log2FC (fold change) > 1 and *P* < .05. The cases from TCGA database were used as the training set. First, we matched the IRG list with the results of DEGs, thus obtaining the differentially expressed IRGs. Then, we performed univariate Cox regression analysis to determine the relationship between patient survival and IRG expression. IRGs with *P* < .05 were selected for multivariate Cox regression analysis, by which a model was built to predict the risk score of each patient. An optimal cutoff was elaborated by the slope variation of the risk score curve. More specifically, the cutoff point with the most obvious slope change in the risk score curve was used as the threshold for classification. Based on the characteristics of its asymmetric distribution, all patients could be divided into the high-risk, medium-risk, and low-risk groups. We conducted multivariate Cox analysis to test whether the IRG model was independent of clinical characteristics, including age, gender, and pathologic stages. Meanwhile, the area under the curve (AUC) of the ROC curve showed the prognostic ability of the IRG model.

### 2.4. The Relationship between the Methylation Level and the mRNA Expression Level of Prognostic IRGs

Based on the methylation file and the RNA-seq data acquired from TCGA database, we conducted an analysis of the correlation between the methylation and mRNA expression by the cor function of R software. The Kaplan-Meier curves were generated to graphically demonstrate OS difference between the hypermethylation and hypomethylation levels of IRGs. An optimal cutoff was elaborated by an iterative approach (68.2%), stratifying patients into mPITX3 hypermethylated (mPITX3 high) and hypomethylated (pPITX3 low) cases.

## 3. Results

### 3.1. Differentially Expressed mRNAs and IRGs in Patients with LUAD

**Figure 1:**
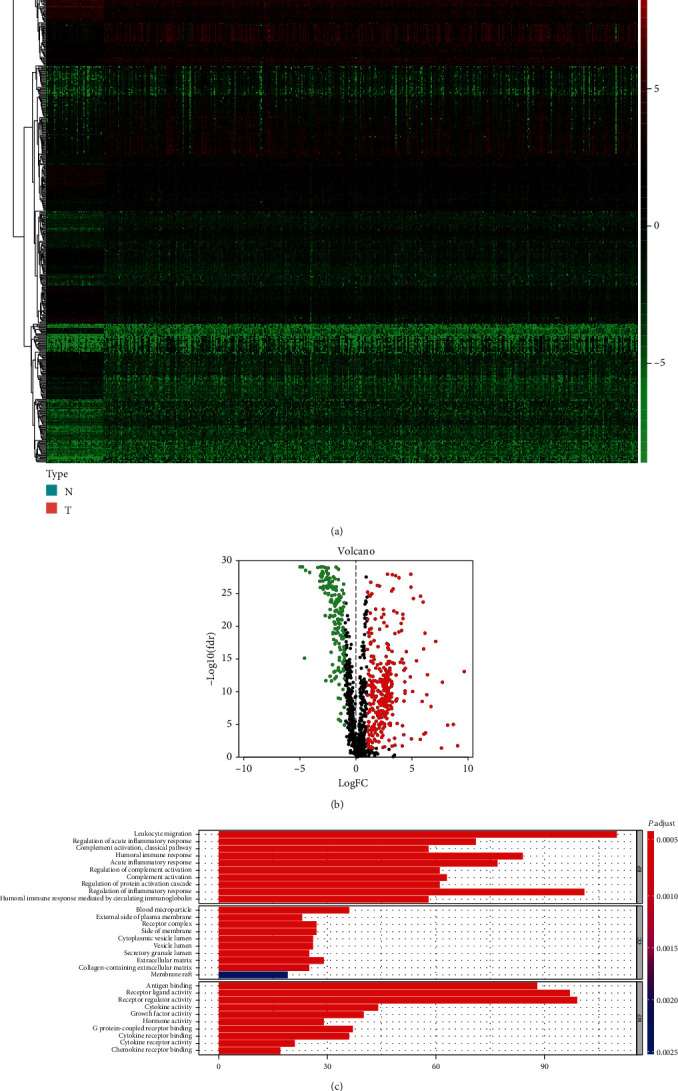
(a) Heatmap of 488 differentially expressed IRGs. The red color indicated the higher gene expression values while the green color indicated the lower gene expression values. N indicated nontumor tissues. T indicated tumor tissues. (b) Volcano plot of 488 differentially expressed IRGs. (c) Bar plot of functional enrichment analyses. BP: biological process; CC: cellular component; MF: molecular function.

Analyses of mRNA expression profiles between adjacent normal tissues and LUAD tissues identified 6729 DEGs in total. Compared with normal lung samples, 1639 mRNAs were downregulated and 5090 were upregulated in LUAD samples. After matching with 2499 IRGs obtained from the ImmPort database, we got 488 differential IRGs, which included 325 downregulated genes and 163 upregulated genes (Figures 1(a) and 1(b)).

### 3.2. Functional Annotation of the IRGs

Enrichment analysis of the differential IRGs offered a biological understanding and identified 10 overrepresented biological processes in gene GO term functional enrichment (Figure 1(c)). Most biological processes were leukocyte migration, regulation of acute inflammatory response, and complement activation. In molecular function, these genes were shown to be notably associated with antigen binding, receptor ligand activity, and receptor regulator activity.

### 3.3. Evaluation of the Prognostic IRGs with TCGA Dataset

**Figure 2:**
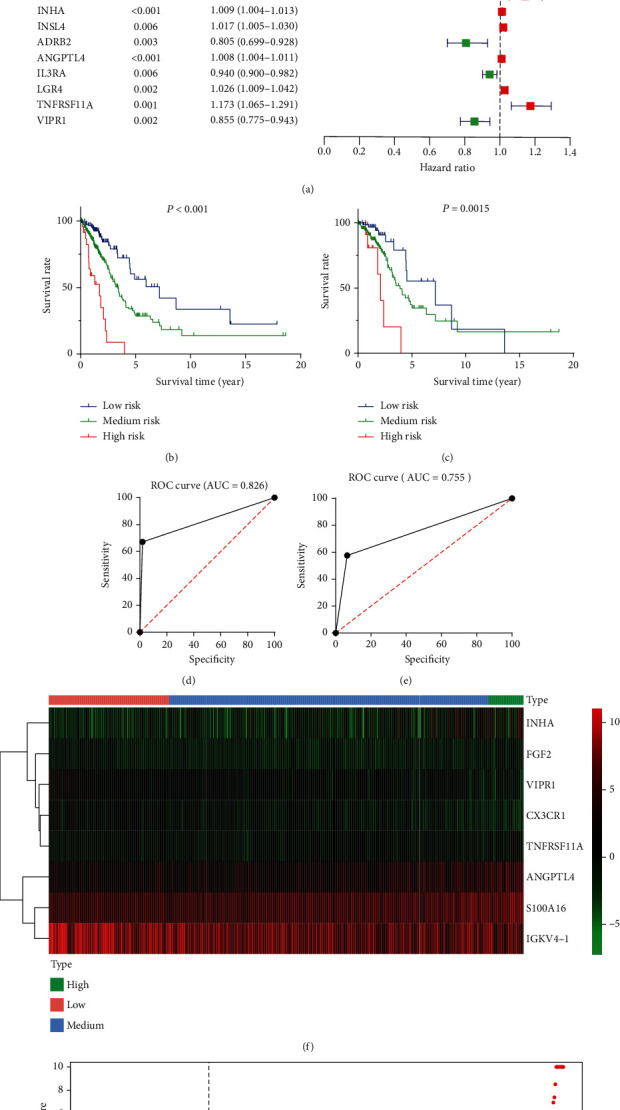
(a) Univariate Cox regression analysis indicated 25 prognostic IRGs. (b) The Kaplan-Meier plot demonstrated that low-risk patients had a longer OS than medium-risk and high-risk patients. (c) Survival analysis of patients in pathological stages 1 and 2 suggests that the IRG model is effective in the early stage LUAD patients. (d, e) The five-year and three-year ROC curves showed that the AUC of the IRG prognostic model are 0.826 and 0.755. (f) Heatmap of eight prognostic IRGs. (g) Patients with LUAD were divided into the high-risk, medium-risk, and low-risk groups depending on the IRG model.

To identify prognosis-specific IRGs that were related to the survival of LUAD patients, univariate Cox regression analysis of 488 differential IRGs was performed. We selected a candidate IRG pool at a *P* < .05 significance threshold. Then, a total of 25 candidate IRGs were subjected to multivariate Cox regression analysis to screen the independent prognostic IRGs (Figure 2(a)). Finally, we obtained the expression coefficients of 8 independent IRGs by multivariate Cox regression analysis. A formula of risk score was generated as follows:

$$\begin{array}{c} \text{risk value}=\left(0.0016\times \text{S}100\text{A}16\text{ expression}\right)+\left(0.2570\times \text{FGF}2\text{ expression}\right)+\left(‐0.0003\times \text{IGKV}4‐1\text{ expression}\right)+\left(‐0.0984\times \text{CX}3\text{CR}1\text{ expression}\right)+\left(0.0079\times \text{INHA expression}\right)+\left(0.0057\times \text{ANGPTL}4\text{ expression}\right)+\left(0.1793\times \text{TNFRSF}11\text{A expression}\right)+\left(‐0.1091\times \text{VIPR}1\text{ expression}\right). \end{array}$$

The risk score for each patient was calculated, and all patients were divided into the low-risk, medium-risk, and high-risk groups according to the changes in the slope of the risk score curve. We choose the cutoff point when the curve slope changes the most. Specifically, the risk score less than 0.7 is considered as low risk. Patients with the risk score between 0.7 and 2.4 were grouped as medium risk, and patients with the risk score greater than 2.4 were divided into the high-risk group (Figures 2(f) and 2(g)). Low-risk patients had a longer OS than medium-risk and high-risk patients (*P* < .001; Figure 2(b)). The five-year and three-year ROC curves showed that the AUC of the IRG prognostic model are 0.826 and 0.755 (Figures 2(d) and 2(e)), indicating that the model has high sensitivity and specificity to predict the prognosis of LUAD patients.

### 3.4. Confirmation of IRGs Expression Patterns via Principal Component Analysis (PCA)

**Figure 3:**
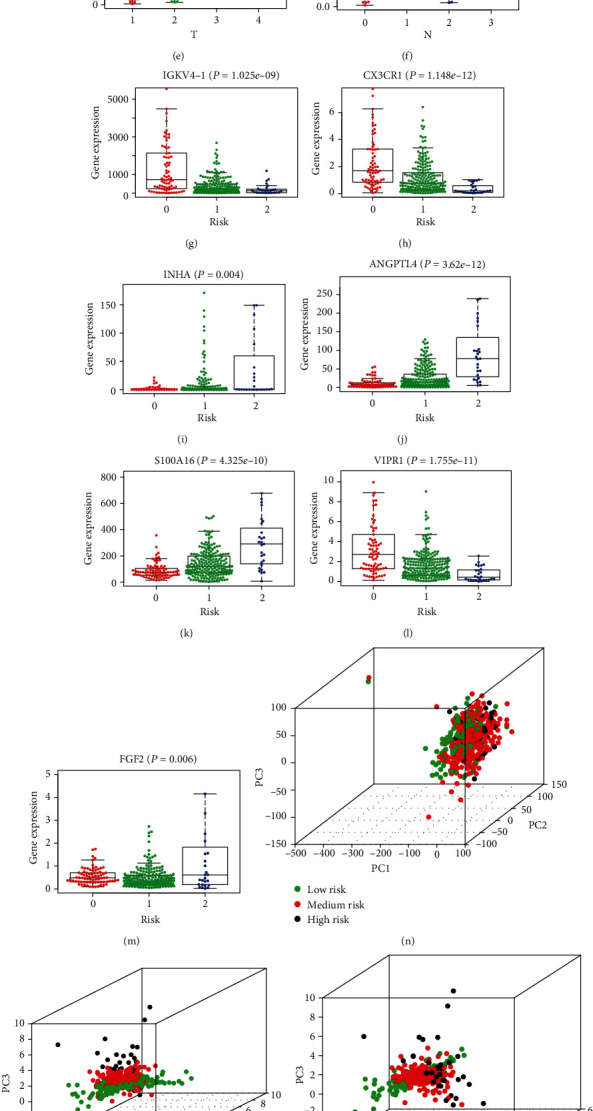
(a, b) The correlation analyses between immune cells and the risk score showed that the contents of B cells and CD4 T cells in tumor tissues decreased with the rise of risk value. (c) The risk score was associated with survival outcome. “fustat = 0” represents “alive,” “fustat = 1” represents “dead.” (d–f) The risk score was significantly associated with pathological stages, T stages, and N stages. (g–m) IGKV4-1, CX3CR1, INHA, ANGPTL4, S100A16, VIPR1, and FGF2 showed different expression levels in the high-risk, medium-risk, and low-risk groups. “risk = 0” represents low risk. “risk = 1” represents medium risk. “risk = 2” represents high risk. (n) PCA depending on all genes from TCGA cohort. (o–q) PCA depending on the IRG model.

First, we conducted PCA depending on all genes from the TCGA cohort, revealing ambiguous distribution patterns between different groups (Figure 3(n)). Then, PCA depending on the IRG model showed significantly different distribution patterns from three directions (Figures 3(o)–3(q)), suggesting that our IRG model can distinguish LUAD patients effectively.

### 3.5. The Relationships of Risk Score with Immune Cells

To comprehensively investigate tumor-immune interactions and to explore which immune cells (including B cells, CD4 T cells, CD8 T cells, neutrophil cells, macrophage cells, and dendritic cells) are associated with the risk score [12], correlation analyses between immune cells and the risk core were performed. The tumor-infiltrating immune subsets for TCGA cohort were downloaded from the Tumor Immune Estimation Resource (TIMER; http://cistrome.shinyapps.io/timer) database. The immune cells with *P* values <.05 were considered to be associated with the investigated genes. As a result, B cells and CD4 T cells were correlated with the risk score (Figures 3(a) and 3(b)). Specifically, the contents of B cells and CD4 T cells in tumor tissues decreased with the rise of the risk score. Since the absolute value of the correlation coefficient is less than 0.5, we consider the correlation between the risk score and B cells or CD4 T cells to also confound other factors. Based on the result of the GO analysis, the complement system is also involved in the action of immune genes.

### 3.6. Relationship between IRG Model and Clinical Parameters

To further understand the relationship between the IRG model and other clinical data, chi-square test and Wilcoxon test analyses were performed to explore the associations between clinical parameters and the risk scores. The results showed that the risk score was significantly associated with pathological stages (*P* = .002), T stages (*P* < .001), N stages (*P* < .001), and survival outcome (*P* = .036) (Figures 3(c)–3(f)). Analysis of variance (ANOVA) also showed statistical differences in the risk scores between the four groups of pathological stages (*P* = .0012), T stages (*P* < .0001), and N stages (*P* = .0009), showing statistically significant differences in each boxplots. From Figure 3(d), it can be seen that the risk score increases with the increase in staging, suggesting that patients with an advanced pathological stage have a higher risk score. But the median risk scores of T4 and N3 patients do not reach the highest as expected, based on Figures 3(e) and 3(f). We consider that it is due to the limited number of samples in T4 and N3 patients, especially since there is only one patient in N3. On the other hand, it may also suggest that our risk assessment system is more effective in early stage LUAD patients. Therefore, we performed survival analysis of patients in pathological stages 1 and 2 alone, and the results suggest that our scoring system is valid in the early stage LUAD patients (Figure 2(c)). Of the eight IRGs, IGKV4-1, CX3CR1, INHA, ANGPTL4, S100A16, VIPR1, and FGF2 showed differential expression levels in the high-risk, medium-risk, and low-risk groups (Figures 3(g)–3(m)).

### 3.7. The Methylation Levels of INHA and S100A16 Impact Its mRNA Expressions and Patient Survival

**Figure 4:**
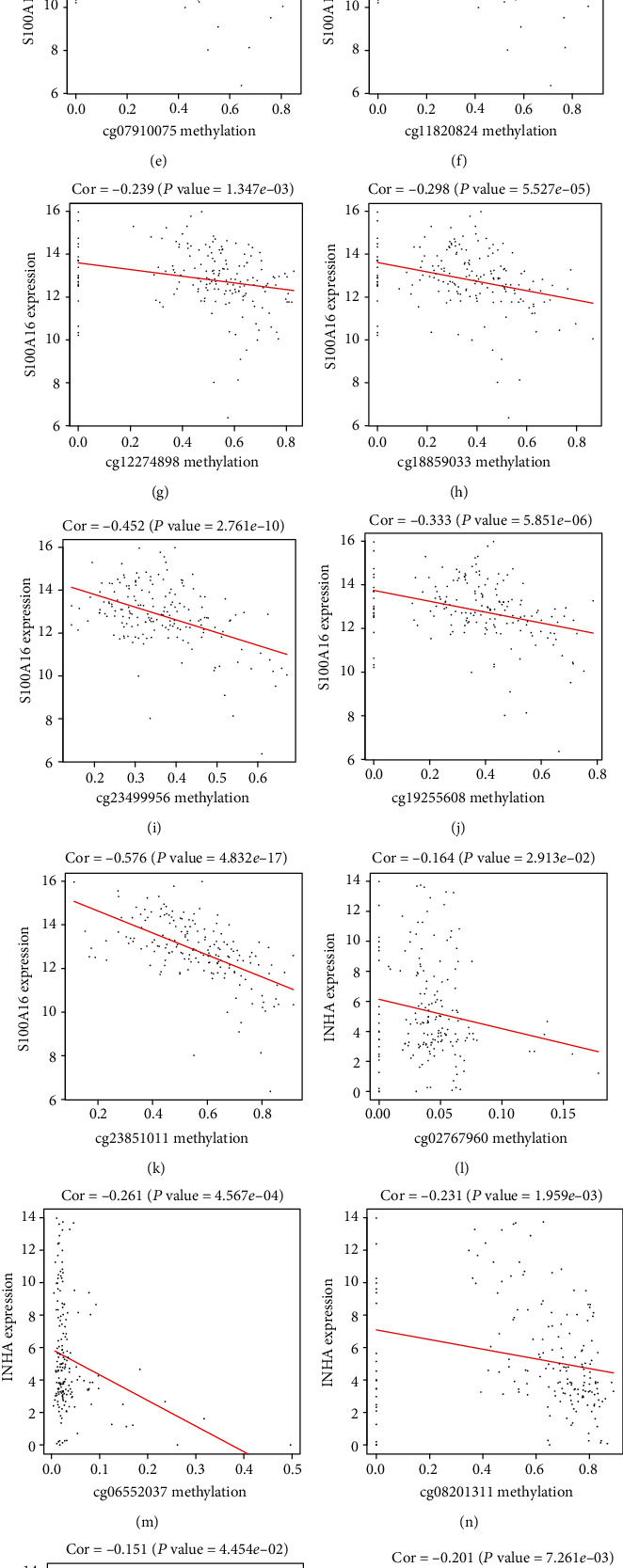
(a, b) The expressions of INHA and S100A16 were negatively correlated with its methylation levels. (c–k) There were 9 CpG sites (cg04990202, cg06213626, cg07910075, cg11820824, cg12274898, cg18859033, cg19255608, cg23499956, and cg23851011) of S100A16 that showed a negative correlation between methylation level and expression level. (l–q) There were 6 CpG sites (cg02767960, cg06552037, cg08201311, cg08493959, cg13858106, and cg22472148) of INHA that showed a negative correlation between methylation level and expression level.

**Figure 5:**
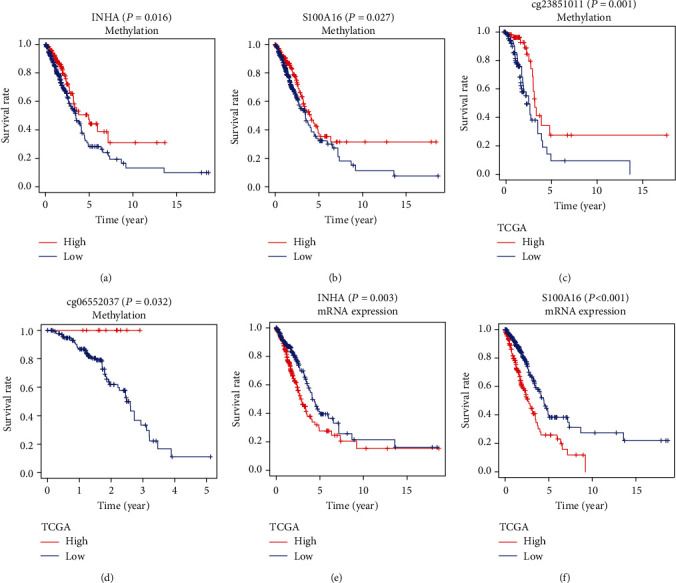
(a, b) The Kaplan-Meier curves showed that patients with hypermethylation levels of INHA and S100A16 tended to have a better OS. (c, d) The Kaplan-Meier analysis pointed out that cg23851011 of S100A16 and cg06552037 of INHA were positively related to survival in the TCGA dataset. (e, f) A better survival rate was shown in the patients with lower mRNA expressions of INHA and S100A16 in the TCGA dataset.

**Figure 6:**
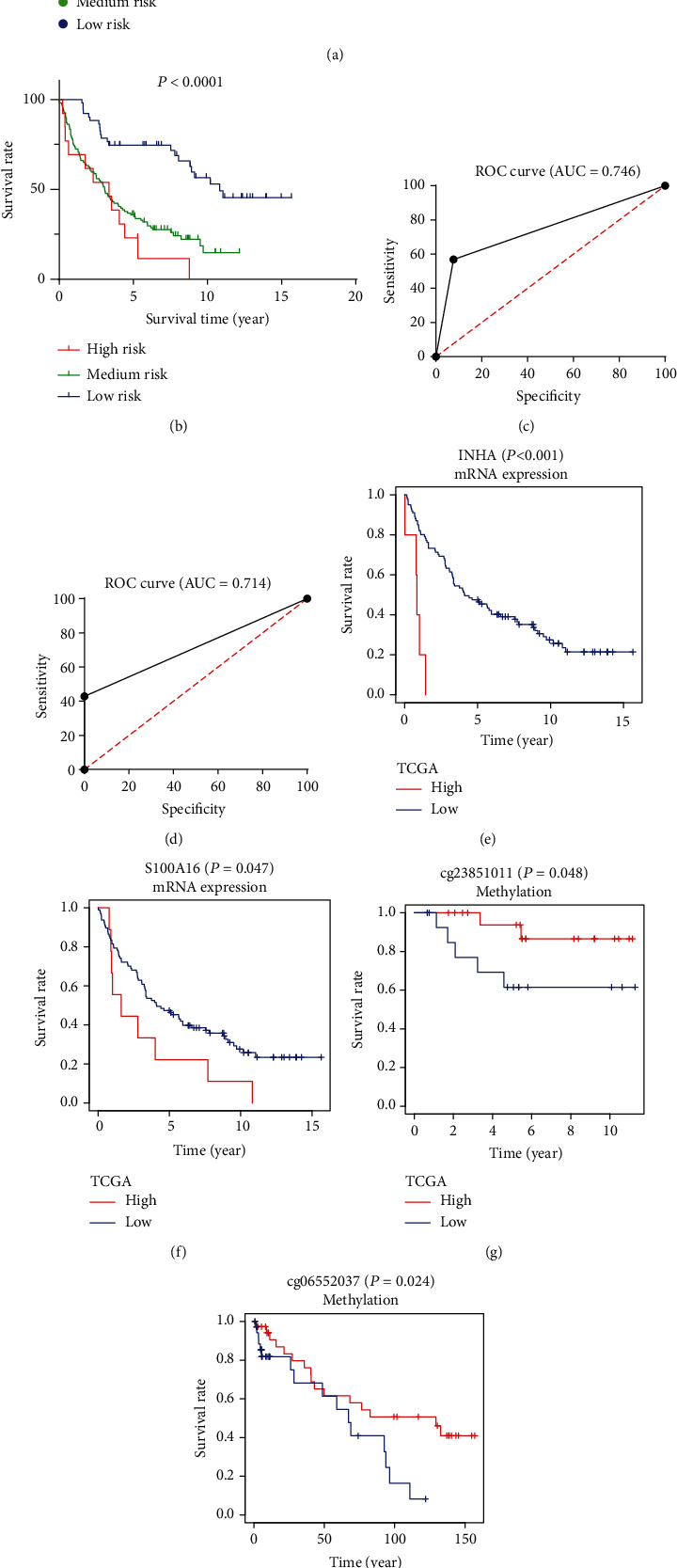
(a) Patients with LUAD were divided into the high-risk, medium-risk, and low-risk groups depending on the IRG model in the GSE37745 dataset. (b) The Kaplan-Meier analysis confirmed that patients with low-risk scores had markedly longer survival rate than high-risk and medium-risk patients in the GSE37745 dataset. (c, d) The five-year and three-year ROC curves showed that the AUC of the IRG prognostic model are 0.746 and 0.714. (e, f) A better survival rate was shown in the patients with lower mRNA expressions of INHA and S100A16 in the GSE37745 dataset. (g, h) The Kaplan-Meier analysis pointed out that cg23851011 of S100A16 and cg06552037 of INHA were positively related to survival in the GSE63384 dataset and the GSE83845 dataset.

Among the eight independent IRGs screened by multivariate Cox regression analysis, the mRNA expressions of INHA and S100A16 were negatively correlated with its methylation levels (Figures 4(a) and 4(b)). Patients with hypermethylation levels of INHA and S100A16 tended to have a better OS (Figures 5(a) and 5(b)). For the exploration of significant CpG sites in LUAD cancer tissues, we analyzed TCGA samples that were profiled using the Illumina Human Methylation 450 platform including 485,577 CpG sites. 9 CpG sites (cg04990202, cg06213626, cg07910075, cg11820824, cg12274898, cg18859033, cg19255608, cg23499956, and cg23851011) of S100A16 showed a negative correlation between methylation and expression level (Figures 4(c)–4(k)), and 6 CpG sites (cg02767960, cg06552037, cg08201311, cg08493959, cg13858106, and cg22472148) of INHA indicated a similar negative correlation (Figures 4(l)–4(q)). But only the absolute value of the correlation coefficient between cg23851011 and S100A16 is greater than 0.5, which may indicate that mRNA expression of IRG is controlled by more complex patterns of the CpG site. Further Kaplan-Meier analysis pointed out that cg23851011 of S100A16 and cg06552037 of INHA were negatively related to survival, which means that higher methylation levels at cg23851011 and cg06552037 lead to better OS (Figures 5(c) and 5(d)). Given that our previous analysis suggested that the methylation levels of these two sites were negatively correlated with the methylation levels of S100A16 and INHA, the results seemed to be contradictory. But considering that the methylation level of each gene is simultaneously influenced by multiple CpG sites, these results also seem to indicate that CpG sites are involved in affecting the prognosis in LUAD through more complex cooperative approaches.

### 3.8. The Validation of the IRG Model via an Independent Cohort

The independent external dataset was utilized for further validation analysis to confirm the robustness of the IRG prognostic model. We calculated the risk score of each patient in GEO dataset GSE37745 (*n* = 196) by the same IRG formula. The patients were also divided into the high-risk, medium-risk, and low-risk groups by the cutoff point depending on the changes in the curve slope (Figure 6(a)). The Kaplan-Meier analysis confirmed the prognostic ability of the IRG model, which showed that patients with lower risk scores had markedly longer OS than medium-risk and high-risk patients (*P* < .0001; Figure 6(b)). The five-year and three-year ROC curves showed that the AUC of the IRG prognostic model are 0.746 and 0.714, respectively (Figures 6(c) and 6(d)). In addition, we further analyzed the survival impact of INHA and S100A16. A better survival rate in the patients with lower mRNA expressions of INHA and S100A16 (Figures 6(e) and 6(f)) was observed, which is consistent with results from TCGA cohort (Figures 5(e) and 5(f)).

### 3.9. The Validation of the Relationship between CpG Sites and Survival via Independent Cohorts

To validate that the differential methylation at the two CpG sites (cg23851011 and cg06552037) would affect clinical outcome, we also performed validation cohorts of 74 LUAD samples and 35 adjacent samples from the GSE63384 dataset and the GSE83845 dataset. The two CpG sites of INHA and S100A16 showed survival differences between high methylation and low methylation levels significantly (Figures 6(g) and 6(h)).

### 3.10. The IRG Model Is an Independent Prognostic Factor for LUAD Patients

**Figure 7:**
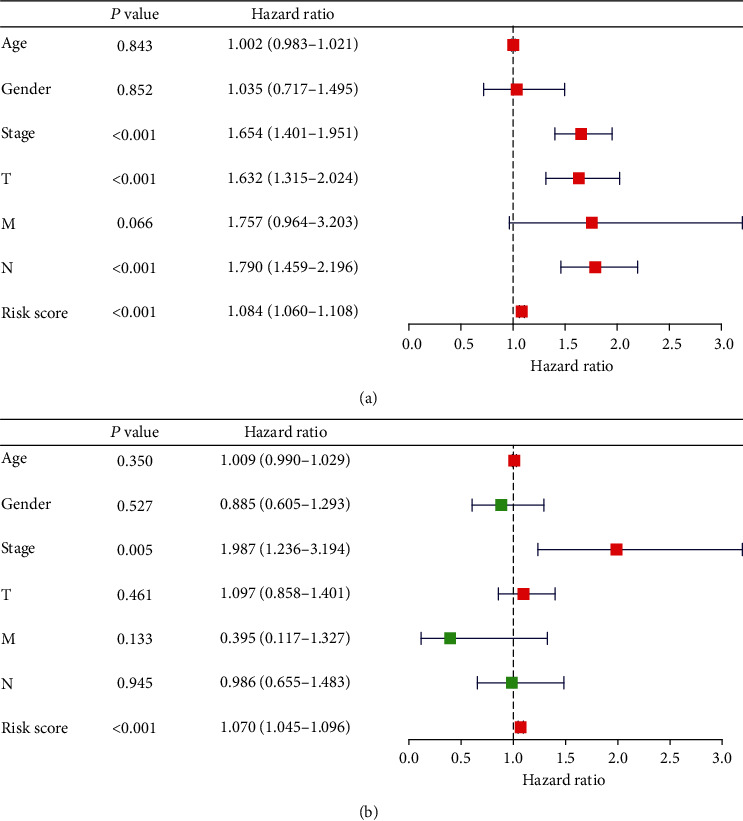
(a) Univariate Cox regression analysis verified the independent predictive value of the IRG model. (b) Multivariate Cox regression analysis verified the independent predictive value of the IRG model.

Univariate Cox regression analysis and multivariate Cox regression analysis were chosen to verify the independent predictive value of the IRG model (Figures 7(a) and 7(b)). Univariate Cox analysis showed that the IRG model, pathological stages, T stages, and N stages were all correlated with OS of LUAD patients. Then, those factors were included in the multivariate Cox analysis, which showed that the IRG model serves as an independent predictive factor.

## 4. Discussion

LUAD, which constitutes approximately 30%-40% of NSCLC, is a global public health problem and the most common cause of cancer-related death [13]. Even after complete surgical resection and chemotherapy, patients with LUAD are still at high risk for recurrence and death. Research and improvement of treatment have shown that the immune system and immune destruction are determining factors during cancer initiation and progression [14, 15]. Recent immunotherapies which target immune checkpoints such as PD-1 have been an alternative treatment and achieved remarkable response in NSCLC [10, 11]. Intratumoral infiltration by immune cells like the cytotoxic lymphocyte has been proven to be related to prognosis in NSCLC [16–18], which is consistent with our result in the GO term. Enrichment analyses of IRGs revealed that LUAD was related to immune processes like leukocyte migration or cytokine interaction.

In this study, we combined multiple gene expression datasets to develop and validate an individualized prognostic signature based on IRGs for LUAD. Gene set enrichment analyses indicated that LUAD was strongly related to 8 IRGs (S100A16, FGF2, IGKV4-1, CX3CR1, INHA, ANGPTL4, TNFRSF11A, and VIPR1). An IRG prognostic model was generated to predict LUAD patient prognosis accurately with high statistical power. Among these eight IRGs, some have been shown to be associated with lung cancer or LUAD, and the role of some in the development of lung cancer remains unclear. The S100 proteins are responsible for cell growth, differentiation, and cell cycle regulation [19]. S100 calcium binding protein A16 (S100A16) is found in a wide spectrum of adult human tissues [20]. Its abnormal expression could be found in lung cancer [21]. Specifically, high S100A16 expression was found to be significantly associated with a poor prognosis in lung cancer [22–24]. FGF2, also known as a basic fibroblast growth factor (FGF), is an important regulator of cell growth and differentiation under physiological and pathological conditions [25, 26]. FGF2 is frequently dysregulated in cancer, especially in advanced stages of hematological and solid tumors, working as a potent proangiogenic growth factor [27]. Immunoglobulin kappa variable 4-1 (IGKV4-1) participates in antigen recognition. Immunoglobulins, also known as antibodies, are membrane-bound or secreted glycoproteins produced by B lymphocytes [28]. In the recognition phase of humoral immunity, the membrane-bound immunoglobulins serve as receptors, which upon binding of a specific antigen, trigger the clonal expansion and differentiation of B lymphocytes into immunoglobulin-secreting plasma cells [29, 30]. The chemokine, fractalkine (FKN), has been discovered and identified in the 1990s, which includes a CX3C chemokine domain and constitutes the CX3C family, including CX3C motif-ligand 1 (CX3CL1) and CX3C motif receptor 1 (CX3CR1) [31, 32]. Researchers have reported that the CX3CL1 and CX3CR1 are enhanced in the pulmonary microvascular system and trigger angiogenesis [33, 34]. INHA catalyzes the NADH-dependent reduction of enoyl-ACP in the biosynthesis of fatty and mycolic acids, which form an essential component of the membrane and cell wall of *M. tuberculosis*, respectively [35, 36]. Most studies focus on its pathway in antituberculosis. Little has been revealed on its affection in tumors. Angiopoietin-like 4 protein (ANGPTL4) is part of the angiopoietin (ANG) superfamily that modulates angiogenesis and is mainly expressed in the liver and adipose tissue [37]. The roles of ANGPTL4 in cancer are still controversial. It is reported to inhibit cell growth, angiogenesis, and metastasis in lung cancer [38]. But studies also showed an opposite function in some other types of cancer [39]. The tumor necrosis factor receptor superfamily member 11a (TNFRSF11A) is the receptor for RANK ligand (RANKL) and part of the RANK/RANKL/OPG signaling pathway that regulates osteoclast differentiation and activation [40]. The vasoactive intestinal peptide receptor-1 (VIPR1) has an important neuropeptide that controls lung physiology and main functions. VIP antagonist in vitro inhibits the proliferation of NSCLC and reduces the growth of NSCLC tumors transplanted into nude mice [41, 42].

DNA methylation represents the most common molecular mechanism of epigenetic modification when genes undergo changes such as cell proliferation or differentiation, alternative splicing, and genetic imprinting [43–45]. Studies have shown that DNA methylation can be closely related to the development of different tumors, thus making methylated genes potential biomarkers for the diagnosis and prognosis of lung cancer [24, 46–48]. A previous study has indicated that the expression level of S100A16 might be modulated by its DNA hypomethylation and serves as an independent prognostic indicator of unfavorable OS and RFS in LUAD [48]. There are studies of methylation levels in other genes that demonstrate the effect of methylation on the prognosis of LUAD. Hypermethylation-mediated downregulation of runt-related transcription factor 3 (RUNX3) could induce docetaxel chemoresistance in LUAD [49]. CDH13 promoter is hypermethylated in LUAD and might be a potential diagnostic biomarker for diagnosis [50].

Among eight prognostic IRGs, INHA and S100A16 showed a correlation between methylation and mRNA expression levels, or methylation levels and survival times. Further detection of CpG sites indicated that higher methylation levels at cg23851011 and cg06552037 led to better OS. At the same time, the verification test in the GEO database also proved the expression levels of INHA and S100A16; methylation levels at cg23851011 and cg06552037 could affect the prognosis of patients with LUAD.

These pieces of evidence support the notion that hypermethylation of INHA and S100A16 leads to gene expression inhibition and better OS, suggesting that INHA and S100A16 could be used as a potential biomarker for prognosis associated with LUAD. The CpG sites cg23851011 and cg06552037 may affect the expression of INHA and S100A16 in a multiple pattern and would be potential markers for treatment. In addition, we established a risk assessment formula for LUAD patients, which is confirmed by an external database. As gene testing is increasingly widely used in clinical practice, this will be another option for clinicians to assess risk.

In summary, molecular mechanisms play an important role in the relationship between IRGs and LUAD. Our results are expected to be applied to clinical practice, which means it may suggest potential targeted therapies for LUAD patients. Further investigations will provide more information of internal mechanisms. Our study reveals that the IRG pattern may affect the prognosis of patients with LUAD. INHA, S100A16, the CpG site cg23851011, and the CpG site cg06552037 may be used as the potential regulators for the treatment of LUAD.

## Data Availability

The datasets supporting the conclusions of this article are available in TCGA database and the GEO database. All of those studies were approved previously by their respective institutional review boards.

## Abbreviations

ALK: Anaplastic lymphoma kinase
ANG: Angiopoietin
ANGPTL4: Angiopoietin-like 4 protein
APCs: Antigen-presenting cells
CX3CL1: CX3C motif-ligand 1
CX3CR1: CX3C motif receptor 1
CTLA-4: Cytotoxic T lymphocyte-associated antigen-4
DEGs: Differentially expressed mRNAs
EGFR: Epidermal growth factor receptor
FGF: Fibroblast growth factor
FKN: Fractalkine
GEO: Gene Expression Omnibus
IRGs: Immune-related genes
ImmPort: Immunology Database and Analysis Portal
IGKV4-1: Immunoglobulin kappa variable 4-1
LUAD: Lung adenocarcinoma
MHC: Major histocompatibility complexes
NSCLC: Non-small cell lung cancer
OS: Overall survival
PCA: Principal component analysis
PD-1: Programmed death 1
RANKL: RANK ligand
RNA-seq: RNA sequencing
ROS1: ROS protooncogene 1
RUNX3: Runt-related transcription factor 3
TCGA: The Cancer Genome Atlas
TIMER: Tumor Immune Estimation Resource
TNFRSF11A: Tumor necrosis factor receptor superfamily member 11a
TKIs: Tyrosine kinase inhibitors
VIPR1: Vasoactive intestinal peptide receptor-1.

## References

[1] Siegel R. L., Miller K. D., Jemal A. (2018). Cancer statistics, 2019. *CA: A Cancer Journal for Clinicians*.

[2] Gray M. E., Meehan J., Sullivan P. (2019). Ovine pulmonary adenocarcinoma: a unique model to improve lung cancer research. *Frontiers in Oncology*.

[3] Hirsch F. R., Scagliotti G. V., Mulshine J. L. (2017). Lung cancer: current therapies and new targeted treatments. *The Lancet*.

[4] Shi J. F., Wang L., Wu N. (2019). Clinical characteristics and medical service utilization of lung cancer in China, 2005-2014: Overall design and results from a multicenter retrospective epidemiologic survey. *Lung Cancer*.

[5] Zhou C., Yao L. D. (2016). Strategies to improve outcomes of patients with EGRF-mutant non-small cell lung cancer: review of the literature. *Journal of Thoracic Oncology*.

[6] Hanna N., Johnson D., Temin S., Masters G. (2017). Systemic therapy for stage IV non-small-cell lung cancer: American Society of Clinical Oncology clinical practice guideline update summary. *Journal of Oncology Practice/American Society of Clinical Oncology*.

[7] Brahmer J., Reckamp K. L., Baas P. (2015). Nivolumab versus docetaxel in advanced squamous-cell non-small-cell lung cancer. *The New England Journal of Medicine*.

[8] Hellmann M. D., Paz-Ares L., Bernabe Caro R. (2019). Nivolumab plus ipilimumab in advanced non-small-cell lung cancer. *The New England Journal of Medicine*.

[9] Beatty G. L., Gladney W. L. (2015). Immune escape mechanisms as a guide for cancer immunotherapy. *Clinical Cancer Research*.

[10] Xu X., Huang Z., Zheng L., Fan Y. (2018). The efficacy and safety of anti-PD-1/PD-L1 antibodies combined with chemotherapy or CTLA4 antibody as a first-line treatment for advanced lung cancer. *International Journal of Cancer*.

[11] Topalian S. L., Hodi F. S., Brahmer J. R. (2012). Safety, activity, and immune correlates of anti-PD-1 antibody in cancer. *The New England Journal of Medicine*.

[12] Li T., Fan J., Wang B. (2017). TIMER: a web server for comprehensive analysis of tumor-infiltrating immune cells. *Cancer Research*.

[13] Ferlay J., Shin H. R., Bray F., Forman D., Mathers C., Parkin D. M. (2010). Estimates of worldwide burden of cancer in 2008: GLOBOCAN 2008. *International Journal of Cancer*.

[14] Angell H., Galon J. (2013). From the immune contexture to the Immunoscore: the role of prognostic and predictive immune markers in cancer. *Current Opinion in Immunology*.

[15] Gentles A. J., Newman A. M., Liu C. L. (2015). The prognostic landscape of genes and infiltrating immune cells across human cancers. *Nature Medicine*.

[16] Galon J., Costes A., Sanchez-Cabo F. (2006). Type, density, and location of immune cells within human colorectal tumors predict clinical outcome. *Science*.

[17] Al-Shibli K. I., Donnem T., Al-Saad S., Persson M., Bremnes R. M., Busund L. T. (2008). Prognostic effect of epithelial and stromal lymphocyte infiltration in non-small cell lung cancer. *Clinical Cancer Research*.

[18] Galon J., Pagès F., Marincola F. M. (2012). Cancer classification using the Immunoscore: a worldwide task force. *Journal of Translational Medicine*.

[19] Donato R. (2003). Intracellular and extracellular roles of S100 proteins. *Microscopy Research and Technique*.

[20] Babini E., Bertini I., Borsi V. (2011). Structural characterization of human S100A16, a low-affinity calcium binder. *Journal of Biological Inorganic Chemistry*.

[21] Marenholz I., Heizmann C. W. (2004). S100A16, a ubiquitously expressed EF-hand protein which is up-regulated in tumors. *Biochemical and Biophysical Research Communications*.

[22] Saito K., Kobayashi M., Nagashio R. (2015). S100A16 is a prognostic marker for lung adenocarcinomas. *Asian Pacific Journal of Cancer Prevention*.

[23] Katono K., Sato Y., Kobayashi M. (2017). S100A16, a promising candidate as a prognostic marker for platinum-based adjuvant chemotherapy in resected lung adenocarcinoma. *OncoTargets and Therapy*.

[24] Kobayashi M., Nagashio R., Saito K. (2018). Prognostic significance of S100A16 subcellular localization in lung adenocarcinoma. *Human Pathology*.

[25] Korc M., Friesel R. E. (2009). The role of fibroblast growth factors in tumor growth. *Current Cancer Drug Targets*.

[26] Beenken A., Mohammadi M. (2009). The FGF family: biology, pathophysiology and therapy. *Nature Reviews Drug Discovery*.

[27] Gualandris A., Rusnati M., Belleri M. (1996). Basic fibroblast growth factor overexpression in endothelial cells: an autocrine mechanism for angiogenesis and angioproliferative diseases. *Cell Growth & Differentiation*.

[28] Lefranc M. P. (2014). Immunoglobulin and T cell receptor genes: IMGT® and the birth and rise of immunoinformatics. *Frontiers in Immunology*.

[29] McHeyzer-Williams M., Okitsu S., Wang N., McHeyzer-Williams L. (2011). Molecular programming of B cell memory. *Nature Reviews Immunology*.

[30] Schroeder H. W., Cavacini L. (2010). Structure and function of immunoglobulins. *The Journal of Allergy and Clinical Immunology*.

[31] Bazan J. F., Bacon K. B., Hardiman G. (1997). A new class of membrane-bound chemokine with a CX3C motif. *Nature*.

[32] Jung S., Aliberti J., Graemmel P. (2000). Analysis of fractalkine receptor CX(3)CR1 function by targeted deletion and green fluorescent protein reporter gene insertion. *Molecular and Cellular Biology*.

[33] Zhang J., Yang W., Luo B., Hu B., Maheshwari A., Fallon M. B. (2012). The role of CX3CL1/CX3CR1 in pulmonary angiogenesis and intravascular monocyte accumulation in rat experimental hepatopulmonary syndrome. *Journal of Hepatology*.

[34] Lee S. J., Namkoong S., Kim Y. M. (2006). Fractalkine stimulates angiogenesis by activating the Raf-1/MEK/ERK- and PI3K/Akt/eNOS-dependent signal pathways. *American Journal of Physiology. Heart and Circulatory Physiology*.

[35] Quemard A., Mazeres S., Sut A., Laneelle G., Lacave C. (1995). Certain properties of isoniazid inhibition of mycolic acid synthesis in cell-free systems of M. aurum and M. avium. *Biochimica et Biophysica Acta (BBA) - Lipids and Lipid Metabolism*.

[36] Quemard A., Sacchettini J. C., Dessen A. (2002). Enzymic characterization of the target for isoniazid in Mycobacterium tuberculosis. *Biochemistry*.

[37] La Paglia L., Listì A., Caruso S. (2017). Potential role of ANGPTL4 in the cross talk between metabolism and cancer through PPAR signaling pathway. *PPAR Research*.

[38] Okochi-Takada E., Hattori N., Tsukamoto T. (2014). ANGPTL4 is a secreted tumor suppressor that inhibits angiogenesis. *Oncogene*.

[39] Liao Y. H., Chiang K. H., Shieh J. M. (2017). Epidermal growth factor-induced ANGPTL4 enhances anoikis resistance and tumour metastasis in head and neck squamous cell carcinoma. *Oncogene*.

[40] Guo L., Elcioglu N. H., Karalar O. K. (2018). Dysosteosclerosis is also caused by TNFRSF11A mutation. *Journal of Human Genetics*.

[41] Moody T. W., Zia F., Draoui M. (1993). A vasoactive intestinal peptide antagonist inhibits non-small cell lung cancer growth. *Proceedings of the National Academy of Sciences of the United States of America*.

[42] Szilasi M., Buglyo A., Treszl A., Kiss L., Schally A. V., Halmos G. (2011). Gene expression of vasoactive intestinal peptide receptors in human lung cancer. *International Journal of Oncology*.

[43] Wilson C. L., Mann D. A., Borthwick L. A. (2017). Epigenetic reprogramming in liver fibrosis and cancer. *Advanced Drug Delivery Reviews*.

[44] Lev Maor G., Yearim A., Ast G. (2015). The alternative role of DNA methylation in splicing regulation. *Trends in Genetics*.

[45] Bestor T. H., Edwards J. R., Boulard M. (2015). Notes on the role of dynamic DNA methylation in mammalian development. *Proceedings of the National Academy of Sciences of the United States of America*.

[46] Harada H., Miyamaoto K., Kimura M., Ishigami T., Taniyama K., Okada M. (2019). Lung cancer risk stratification using methylation profile in the oral epithelium. *Asian Cardiovascular & Thoracic Annals*.

[47] Kemp Jacobsen K., Johansen J. S., Mellemgaard A., Bojesen S. E. (2019). AHRR (cg05575921) methylation extent of leukocyte DNA and lung cancer survival. *PLoS One*.

[48] Chen D., Luo L., Liang C. (2018). Aberrant S100A16 expression might be an independent prognostic indicator of unfavorable survival in non-small cell lung adenocarcinoma. *PLoS One*.

[49] Zheng Y., Wang R., Song H. Z., Pan B. Z., Zhang Y. W., Chen L. B. (2013). Epigenetic downregulation of RUNX3 by DNA methylation induces docetaxel chemoresistance in human lung adenocarcinoma cells by activation of the AKT pathway. *The International Journal of Biochemistry & Cell Biology*.

[50] Pu W., Geng X., Chen S. (2016). Aberrant methylation of CDH13 can be a diagnostic biomarker for lung adenocarcinoma. *Journal of Cancer*.

